# Vital need to engage the community in HIV control in South Africa

**DOI:** 10.3402/gha.v8.27450

**Published:** 2015-08-07

**Authors:** Stefan Hanson, Yanga Zembe, Anna Mia Ekström

**Affiliations:** 1 Department of Public Health Sciences, Karolinska Institutet, Stockholm, Sweden; 2 Health Systems Research Unit, Medical Research Centre, Cape Town, South Africa; 3 Department of Infectious Diseases, Karolinska University Hospital, Stockholm, Sweden

**Keywords:** HIV/AIDS, HIV incidence, South Africa, multiple sexual partnerships, risk groups, TasP, analytical framework, community involvement

## Abstract

According to the latest 2014 UNAIDS report, which was based on the 2012 South African National HIV Prevalence, Incidence & Behavior Survey, there were between 6.3 and 6.4 million HIV infected people in South Africa. Although the number of new infections appears to have declined in the past 5 years, 370,000 new infections were still estimated to occur in 2013. Young, black women were most at risk with a very high incidence of 4.5%. Of the infected, only 2.2 million were on antiretroviral therapy (ART), meaning that the majority living with HIV was not virally suppressed and thus at risk of infecting somebody else. Eight out of 10 South Africans still believed they were at low risk of HIV infection. Condom use was declining and multiple sexual partnerships were increasing. These findings raise questions about whether current control efforts are properly addressing the drivers of the epidemic. Recent behavior change campaigns target intergenerational sex and blame the high transmission rates among girls on ‘sugar daddies’ thus diverting attention away from common risk behaviors in the general population. Reduction of new infections is crucial. Much of the current global HIV debate focuses on treatment as prevention (TasP) – an approach hampered by resource problems and the fact that most people are infected by someone who is unaware of his/her HIV status. This raises doubts TasP alone is a sufficient and sustainable solution to prevention. It is not enough to mainly treat those already infected; there is also a need to allocate more resources to address the root causes – ART plus norm and behavior change. We thus propose increased attention to common sexual and social norms and behaviors. New and harmful community norms are one of the major drivers of the ongoing spread of HIV among young women and men in black communities. Addressing sexual risk behaviors and the gender and sexual norms that influence them to scale requires ensuring communities are provided with skills to reflect on the individual and social mechanisms by which these risk behaviors are generated and normalized. To achieve this, partnerships must be formed between political leaders, researchers, technocrats and affected communities. Considering the severity of the epidemic and the continued high incidence of HIV, it is high time to review the current strategy to HIV control in South Africa and allocate more resources to approaches that emphasize community driven norm and behavior change.

At the launch of the South African National HIV Prevalence, Incidence & Behavior Survey 2012, on April 1, 2014, a number of findings of importance for planning of future HIV control efforts were presented. According to the latest 2014 UNAIDS report based on the survey (1), there were 6.3–6.4 million HIV infected; new infections were still high at 370,000 and 2.2 million people were on antiretroviral therapy (ART). Young black women (aged 20–34) had an extremely high HIV incidence (4.5%), contributing to a high HIV prevalence among women in the 15–49 year age group (23.2% vs. 14.5% in males). Encouragingly incidence was declining among younger women (aged 15–24). In spite of substantial control efforts, 8 out of 10 South Africans still believed they were at low risk of HIV infection. Condom use was declining across all age groups (except above 50 years), and there was an increase in multiple sexual partnerships (2).

These findings prompt the central question if current control efforts in South Africa are the most effective and really address the drivers of the epidemic.

## Drivers of the epidemic

Considerable research efforts have been made to describe and analyze the HIV epidemic in South Africa. Already in 2006, international and African scientists and politicians gathered to identify and discuss the main drivers of the epidemic in Southern Africa, at high-level forums and think tanks (3). The drivers identified included multiple concurrent sexual partnerships, gender power inequities, high population mobility, and cultural norms and belief systems. The consensus among many scholars on these drivers of the HIV epidemic is that the social context in high-prevalence communities in South Africa plays a major role in the way in which sexual risk-taking is configured in the country. In his book ‘Love in the time of AIDS’, Mark Hunter (4) explores transformation of gender and intimacy norms to understand the roots of the extremely high incidence of HIV in South Africa. Through close engagement with an informal settlement where he lived for extended periods of time, collecting love letters, cell phone text messages, oral histories, and archival materials, Hunter highlights the role played first by apartheid and then by social factors such as chronic unemployment in the creation of new and harmful community norms of femininity, masculinity, love, and sex. These have resulted in an economy of exchange that fuels the spread of HIV among young women and men in black communities.

Hunter's findings are echoed by other research studies conducted in South Africa such as those of Leclerc-Madlala and Jewkes & Morrell (5–7). Recently, the findings of another study on sexual risk-taking among young women in a poor community in Cape Town suggested that the context within which high-risk sexual behaviors are negotiated and enacted is characterized by a social environment that prioritizes social belonging, active pursuit of male sexual partners for financial and sexual benefits, while deprioritizing and trivializing the risk of contracting HIV (8).

Additional to the academic efforts to understand the drivers of the epidemic in high-prevalence communities, non-governmental organizations have engaged in HIV prevention and gender norm transformation. Notable among these are Soul City (9) and SONKE Gender Justice (10); these institutions have done substantial work to address multiple concurrent partnerships, gender inequities, and intimate partner violence. In their work they have used social change models, curriculum-based community engagement strategies, media, and information campaigns. These are all laudable efforts, but sexual risk behaviors and the related social norms take time to change. Therefore, other participatory methods may need to be employed, their focus broadened and strengthened by contributions from a wider audience of researchers, implementers, and policy makers. Individual approaches focused on changing individual behaviors, such as those of SONKE Gender Justice (10) and the Stepping Stones Intervention (11), might be effectively combined with community-oriented approaches aimed at changing not only behaviors but also the norms underlying them.

One key actor, the South African Ministry of Health (MoH), has been widely recognized for the major transformation of the South African government's focus and strategy on HIV, overcoming the period of denials and passiveness that characterized the 1990s and early 2000s. However, there are still a number of areas of improvement. The first concerns allocations of funds to the different HIV programs. Currently, government funding is disproportionately skewed in favor of HIV & TB testing and treatment as prevention (TasP) to the neglect of non-medical modalities such as interventions targeting behavior change or social norms. Although in the National Strategic Plan for HIV, TB, and STIs (2012–2016) (12), interventions to this end, such as efforts ‘to change cultural and social norms that increase vulnerability to HIV’; behavioral interventions, such as ‘reducing multiple and concurrent partnerships’ are well mentioned they receive very limited funding. Thus, a recent costing analysis commissioned by the South African National AIDS Council (13) examined the contribution to the total cost of the response of each main program over the 5-year period. The findings were that HIV and TB testing and treatment interventions comprised 85% of the total cost of the response, leaving very little room for social norm change interventions.

The second issue has to do with the misdirection of HIV control efforts. Recently, the provincial government of KwaZulu-Natal, the province with the highest prevalence of HIV in the country, initiated a massive multimedia behavioral change campaign targeting intergenerational sexual partnerships despite the evidence of recent study findings from the Africa Centre indicating that intergenerational sex is not a driving factor behind new HIV infections among young women in KwaZulu-Natal (14). Further, the risk group approach has since long played out its role in South Africa. Emphasizing the role of sugar daddies and sex workers in this generalized epidemic context, where the purchasing of formal sex work is not common (15), diverts attention away from actual drivers of the epidemic including common and widespread HIV-risk behaviors in the general population.

## TasP in South Africa

Much of the current global HIV debate focuses on TasP. Given the fact that one in every six people in the world, who are on ART, reside in South Africa (2), it is important that the potential for TasP in the country is thoroughly analyzed. It is estimated that in some settings 50% of all infections are transmitted by someone who is newly infected, and up to 80% by someone who does not know he/she is infected (16). In South Africa, as much as 62% of HIV-positive men and 45% of women were not aware of their HIV status in 2012 (2). For TasP to have a substantial impact on HIV incidence, early diagnosis and testing is key, but the annual number of people testing for HIV reportedly declined in South Africa from 13 million between 2010 and 2011 to 8.9 million in 2012–2013 (17). Further, only one in three estimated to be living with HIV was on ART, and, although this may be considered a relatively high treatment coverage (18) and was estimated to have reduced incidence by 17–32% in 2011 (19), two out of three living with HIV in South Africa were not virally suppressed and at high risk of infecting somebody else. The high incidence also implies an ever-increasing number of young people in need of life-long ART – adding a substantial burden on the health system. Further expansion to universal access would, according to a South African Special Report, both be ‘logistically untenable and would likely cannibalize the whole health budget’ (20).

## How to tackle the epidemic

This raises doubts that the HIV testing and TasP approaches alone are a sufficient and sustainable solution for ending the HIV endemic in South Africa. Efforts to reduce new infections are crucial. To strengthen these it is not enough to mainly treat those already infected but necessary to also allocate more resources to address the root causes of the epidemic – we therefore suggest HIV testing and early ART initiation are combined with efforts aimed at primary prevention where change of existing sexual and social norms and behaviors is emphasized.

There is sufficient evidence that continued risky behaviors are related to existing norms that condone multiple sexual partnerships (2, 21). Most people will not perceive their own behavior as risky if it is founded on deeply entrenched and popular norms. Addressing these could effectively impact the epidemic if it is done through multiple concerted efforts and at scale. Here we stress the importance of social norms for several reasons. First, sexual risk behavior is shaped by factors beyond what can be controlled by the individual or cognitive decision making (6, 22–24); it is not merely the product of physiological impulses or individual motivations, but rather fashioned by a host of situational factors. These include the limitations and/or opportunities for sexual risk-taking that are provided by the immediate physical environment within which sexual behavior is negotiated. Other important factors that influence sexual behavior include historical, cultural factors and social norms and patterns that provide the codes of behavior for the population at risk (22, 25, 26). Second, sexual risk-taking is not merely affected by broad and generic social norms; it is produced (among other things) by specific, harmful gender norms that predominate in many of the high-prevalence communities in the country (27). Thus, many populations here are caught up in high-risk sexual relationships that are defined by gender and relationships norms that reinforce patriarchy, gender power inequities, and ultimately intimate partner violence, all of which have the effect of increasing the risk of HIV infection (6, 28, 29). Third, many behavioral and biomedical models of HIV prevention are repeatedly shown to have little to no lasting impact, owing to their failure to address and transform the broader social context within which sexual risk behaviors are produced and negotiated (25, 30).

It is important to stress that by highlighting the importance of addressing social norms, we are by no means advocating for a magic bullet or one-size-fits-all HIV prevention strategy. The evidence clearly argues for a mix of strategies that address the epidemic from various angles, targeting the individual, the immediate social environment influencing his or her behaviors as well as the macro sphere within which societies negotiate life (25). Such strategies should be community based, comprising a range of interventions, as long as they are underlined by the recognition that meaningful and lasting behavior change can only happen if the affected populations are adequately capacitated and supported to exercise control over their individual behaviors and the social environment that produces them (31). Further, norm change, may, from a public health perspective, be the only feasible approach as it can be brought to scale. It may also become more effective if combined with small-scale individual behavior change approaches in selected high-prevalence areas, such as risk reduction and HIV prevention interventions.

Here UNAIDS's recognition of the capacity of community systems ‘to organize for their own change’ is supportive (32). This needs be translated into practice and community involvement transformed from an activity mainly undertaken by non-governmental organizations (NGOs) to a central government mechanism by which affected communities are gradually engaged in HIV control efforts aiming at incidence reduction – combining social norm analysis and transformation leading to a depopularization of multiple sexual partnerships, increase in HIV testing – especially among men – and retention on ART. Such efforts could generate what the former president of South Africa, Nelson Mandela, termed ‘a new social revolution’ (33).

To effectively engage communities in such activities there is need for a well-defined analytical framework that brings together the different factors that lead to new HIV infections, providing affected populations with an understanding of the linkages between structural, biological, behavioral and contextual factors and how these relate to the risk behaviors common among them and the social and sexual norms underlying those practices. Such a framework would facilitate the description of the epidemic at the local community level and guide control efforts so that these are implemented in a targeted, locally appropriate and systematic fashion. Uchudi et al. (34) conducted a multilevel analysis to understand the determinants of high-risk sexual behavior, in particular multiple sexual partnering in Sub-Saharan Africa. The multilevel analysis borrowed from ecological models to examine how factors at the macro (societal conditions including cultural factors), meso (family and household characteristics), micro (individual social and human capital) levels influence multiple sexual partnering and the associated risk behaviors (mainly transactional sex) in high HIV-prevalence communities. Community capacity to conduct similar multilevel analysis of the drivers of key sexual behaviors should be developed. Supplementary analytical frameworks that enable communities to undertake analyses of cultural assets and liabilities to enhance local understanding of how cultural belief systems influence HIV risk-taking are also needed.

Beyond this, it is important that local and national leaders partner with communities and address identified problems and at the same time promote ART scale-up. The universities in South Africa here have an important role to play (35, 36) both in describing the context of sexual behavior and designing systematic and participatory ways of enhancing and sharing knowledge with affected communities and to monitor progress.

## Concluding remarks

Considering the findings of 2012 national survey, it is high time to review current strategy and resource allocation to HIV control in South Africa. It is time to allocate more resources to primary prevention and to engage the community.

We recognize that community involvement strategies by necessity are complex, long-winded, and challenging to implement, evaluate, and find evidence for and that the subject itself is both private and sensitive. This likely influences the reluctance and hesitance both in the international leadership and among national politicians to take on this task. We also fear that the present focus on ART may serve as an ‘alibi’ for political leaders for social inaction. But the magnitude of the HIV epidemic in South Africa and the great potential benefits of norm change requires that scientific and political risks are taken and that new innovative approaches of HIV prevention that could counteract some of the existing information fatigue are employed. Further, local people in villages and townships, who are the ones best placed to influence the rules that govern their social life, have a wealth of knowledge about the social processes that shape the epidemic in their communities; researchers and implementers need to tap into this knowledge capital and enable community capacity to reflect on the implications of existing gender, social, and sexual norms for HIV in their communities. This approach also has the potential to increase uptake of ART. Most of us believed ART scale-up was impossible – it was not. We are convinced norm change is also possible.
